# Supplementary material for: Distribution of spontaneous combustion three zones and optimization of nitrogen injection location in the goaf of a fully mechanized top coal caving face
Source: PLoS One. 2021 Sep 20;16(9):e0256911. doi: 10.1371/journal.pone.0256911 (PMC8452049; doi:10.1371/journal.pone.0256911)
Supplement: S1 File — (DOCX) [file pone.0256911.s001.docx]

In addition to the data already provided in this paper, other data are as follows：

1.The design strike length of working face is 906.6m, the inclined length is 90m, the thickness of coal seam is 5.24~7.30m, and the average thickness is 6.17m. Fully mechanized top-coal caving mining technology is adopted to control the goaf roof with caving method. The mining height of the working face is 2.9 m, the top-coal caving height is 3.3 m, and the mining/caving ratio is 1:1.13. The coal seam is well developed with 1~4 layers of gangue. The gangue is mainly thin mudstone, and the thickness changes little. The coal seam dip Angle is 8°~14 °, with an average of 10 °. The mined coal seam belongs to class ⅱ spontaneous combustion coal seam, the spontaneous ignition period is 21~55 days, and the coal dust explosion index is 45.79%, which is explosive. Measuring points are arranged in the inlet and return air lanes at the same time. The distance between measuring points is 20 m, and there are 3 on each side. The length of the working face is 90m, and the width is 7m; Width of goaf is 90m. The width of the air inlet in the air inlet lane is 3m, and the width of the air inlet in the air return lane is 3m.

2. Monitoring data of oxygen concentration changes with working face propulsion degree

| Speed of propulsion /m | oxygen concentration/% | | | | |
| --- | --- | --- | --- | --- | --- |
|  | 1# | 2# | 3# | 4# | 5# |
| 3 | 20.7 | 20.4 | 20.5 | 20.2 | 20.6 |
| 6 | 20.5 | 20.3 | 20.1 | 19.7 | 20 |
| 10 | 20 | 19.9 | 19.7 | 19.3 | 19 |
| 14.5 | 19.6 | 19.8 | 19.2 | 19 | 18.8 |
| 23.8 | 19.2 | 19.3 | 19.1 | 18.8 | 18.4 |
| 32 | 18.8 | 18.9 | 18.8 | 18.3 | 18.2 |
| 40 | 18.7 | 18.5 | 18.5 | 17.6 | 17.9 |
| 44.7 | 18.4 | 18.2 | 18.3 | 17.2 | 17.6 |
| 49 | 18.2 | 18.1 | 18.1 | 16.8 | 16.4 |
| 52 | 17.9 | 17.8 | 17.6 | 16.3 | 16 |
| 55 | 17.5 | 17.3 | 17 | 15.6 | 15.3 |
| 58 | 16.7 | 16.1 | 16.4 | 14.9 | 14.5 |
| 64 | 15.5 | 15 | 14.9 | 13.8 | 13.2 |
| 69.4 | 13.8 | 13.4 | 13.7 | 12.4 | 11.8 |
| 74.6 | 13.1 | 12.8 | 12.5 | 10.7 | 10.2 |
| 87 | 11.9 | 11.3 | 11.6 | 8.4 | 8.8 |
| 90 | 11.3 | 10.8 | 10.8 | 7.9 | 8 |
| 102 | 9.5 | 9 | 9.3 | 7 | 6.7 |
| 105 | 8.5 | 8.2 | 8.5 | 6.6 | 6.6 |
| 109 | 8 | 7.7 | 7.9 | 6.5 | 6.1 |
| 123.6 | 6.9 | 7.1 | 7.6 | 6.3 | 6 |
| 131.7 | 6.5 | 6.9 | 7.3 | 6 | 5.5 |
| 140.4 | 6.4 | 6.2 | 6.7 | 5.7 | 5.8 |

3. Other parameter values used in numerical simulation：

$\mu\text{=}1.8\times10^{\text{-}5}$ kg·m^-1^·s^-1^；*D*=2.88×10^-5^ m^2^/s；n=0.25；*k*=2.88×10-5 m^2^；*b*_0_=0.0235；*k*_1_=1.5；*M*=4.5m；*M*_1_=0.4m；*k*_p_=1.1~1.5；*r*_o_=0.098mol·m^-3^·s^-1^；

*R*_1_=0.0013 N·s^2^·m^-8^；*W*(CH_4_)=0.12~4.7 mol/（m^3^·s）；*l*=90m；*Q*_0_=3.66 m^3^·h^-1^；

*Q_N_*=400 m^3^·h^-1^.

4. Distribution of oxidized spontaneous combustion zone in goaf with different nitrogen injection position

| Position of nitrogen injection (Distance from crest line) (m) | width (m) |
| --- | --- |
| 10 | 44 |
| 20 | 35 |
| 30 | 32 |
| 40 | 28 |
| 50 | 26 |
| 60 | 28 |
| 70 | 30.6 |

5. The data in the oxygen concentration change curve at each measuring point are as follows

| oxygen concentration/% | Speed of propulsion/m | | |
| --- | --- | --- | --- |
|  | 1# | 2# | 3# |
| 18 | 42 | 45 | 41 |
| 13 | 51 | 51 | 49 |
| 8 | 72 | 74 | 73 |
| 7 | 77 | 76 | 78 |
