# Supplementary material for: Distribution of spontaneous combustion three zones and optimization of nitrogen injection location in the goaf of a fully mechanized top coal caving face
Source: PLoS One. 2021 Sep 20;16(9):e0256911. doi: 10.1371/journal.pone.0256911 (PMC8452049; doi:10.1371/journal.pone.0256911)
Supplement: S2 File — (DOCX) [file pone.0256911.s002.docx]

1.The data set in Fig. 2 has been added to the curve of oxygen concentration with advance degree of working face

| Speed of propulsion /m | oxygen concentration/% | | | | |
| --- | --- | --- | --- | --- | --- |
|  | 1# | 2# | 3# | 4# | 5# |
| 3 | 20.7 | 20.4 | 20.5 | 20.2 | 20.6 |
| 6 | 20.5 | 20.3 | 20.1 | 19.7 | 20 |
| 10 | 20 | 19.9 | 19.7 | 19.3 | 19 |
| 14.5 | 19.6 | 19.8 | 19.2 | 19 | 18.8 |
| 23.8 | 19.2 | 19.3 | 19.1 | 18.8 | 18.4 |
| 32 | 18.8 | 18.9 | 18.8 | 18.3 | 18.2 |
| 40 | 18.7 | 18.5 | 18.5 | 17.6 | 17.9 |
| 44.7 | 18.4 | 18.2 | 18.3 | 17.2 | 17.6 |
| 49 | 18.2 | 18.1 | 18.1 | 16.8 | 16.4 |
| 52 | 17.9 | 17.8 | 17.6 | 16.3 | 16 |
| 55 | 17.5 | 17.3 | 17 | 15.6 | 15.3 |
| 58 | 16.7 | 16.1 | 16.4 | 14.9 | 14.5 |
| 64 | 15.5 | 15 | 14.9 | 13.8 | 13.2 |
| 69.4 | 13.8 | 13.4 | 13.7 | 12.4 | 11.8 |
| 74.6 | 13.1 | 12.8 | 12.5 | 10.7 | 10.2 |
| 87 | 11.9 | 11.3 | 11.6 | 8.4 | 8.8 |
| 90 | 11.3 | 10.8 | 10.8 | 7.9 | 8 |
| 102 | 9.5 | 9 | 9.3 | 7 | 6.7 |
| 105 | 8.5 | 8.2 | 8.5 | 6.6 | 6.6 |
| 109 | 8 | 7.7 | 7.9 | 6.5 | 6.1 |
| 123.6 | 6.9 | 7.1 | 7.6 | 6.3 | 6 |
| 131.7 | 6.5 | 6.9 | 7.3 | 6 | 5.5 |
| 140.4 | 6.4 | 6.2 | 6.7 | 5.7 | 5.8 |

2. The data of the variation of the width of oxidizing spontaneous combustion zone in goaf with the position of nitrogen injection port in Fig. 5 have been given in Table 1 of this paper. It's shaded in yellow.

| Position of nitrogen injection (Distance from crest line) (m) | Starting position of oxidation zone (m) | Termination position of oxidation zone (m) | width (m) |
| --- | --- | --- | --- |
| 10 | 46.5 | 90.5 | 44 |
| 20 | 46 | 81 | 35 |
| 30 | 45.5 | 77.5 | 32 |
| 40 | 44 | 72 | 28 |
| 50 | 44 | 70 | 26 |
| 60 | 44.5 | 72.5 | 28 |
| 70 | 46 | 76.6 | 30.6 |

3. Fig.7 The data in the oxygen concentration change curve at each measuring point are as follows

| oxygen concentration/% | Speed of propulsion/m | | |
| --- | --- | --- | --- |
|  | 1# | 2# | 3# |
| 18 | 42 | 45 | 41 |
| 13 | 51 | 51 | 49 |
| 8 | 72 | 74 | 73 |
| 7 | 77 | 76 | 78 |
