# Supplementary material for: Distribution of spontaneous combustion three zones and optimization of nitrogen injection location in the goaf of a fully mechanized top coal caving face
Source: PLoS One. 2021 Sep 20;16(9):e0256911. doi: 10.1371/journal.pone.0256911 (PMC8452049; doi:10.1371/journal.pone.0256911)
Supplement: Attachment — Submitted filename: Response to Reviewers.docx [file pone.0256911.s004.docx]

Dear editor and reviewers:

Thank you for your letter and the reviewers’ comments on our manuscript entitled " Distribution of Spontaneous Combustion Three Zones and Optimization of Nitrogen Injection Location in the Goaf of a Fully Mechanized Top Coal Caving Face". Those comments are very helpful for revising and improving our paper, as well as the important guiding significance to other research. We have studied the comments carefully and made corrections which we hope meet with approval. The main corrections are in the manuscript and the responds to the reviewers’ comments are as follows (the replies are highlighted in blue ).

I checked the list of all references, and I can guarantee the integrity and correctness of each reference.

**Replies to the reviewers’ comments:**

**Response to reviewers 1**

1.The capitalization in the title of the thesis is wrong, it is recommended to make the following changes: Distribution of Three Spontaneous Combustion Zones and Optimization of Nitrogen Injection Sites in the Goaf of a Fully Mechanized Carving Face.

**Response:** **The original title of the paper was "** **Distribution of Spontaneous Combustion three zones and Optimization of Nitrogen Injection Location in the Goaf of a fully Mechanized top Coal Caving Face", which has been polished by Letpub mechanism. Therefore, I think the translation of the title of the paper does not need to be modified, but the case format of the title is wrong, which has been modified now**
